# Accessibility of Opioid Treatment Programs Based on Conventional vs Perceived Travel Time Measures

**DOI:** 10.1001/jamanetworkopen.2024.0209

**Published:** 2024-02-20

**Authors:** Junghwan Kim, Jinhyung Lee, Thomas A. Thornhill, Julia Dennett, Haidong Lu, Benjamin Howell, Lauretta E. Grau, David A. Fiellin, Robert Heimer, Gregg Gonsalves

**Affiliations:** 1 Department of Geography, College of Natural Resources and Environment, Virginia Tech, Blacksburg; 2 Department of Geography and Environment, Faculty of Social Science, Western University, London, Ontario, Canada; 3 Department of Epidemiology of Microbial Diseases, Yale School of Public Health, New Haven, Connecticut; 4 Public Health Modeling Unit, Yale School of Public Health, New Haven, Connecticut; 5 Departments of Medicine and Emergency Medicine, Yale School of Medicine, New Haven, Connecticut; 6 Program in Addiction Medicine, Yale School of Medicine, New Haven, Connecticut; 7 Department of Health Policy and Management, Yale School of Public Health, New Haven, Connecticut

## Abstract

**Importance:**

Transportation barriers have long been associated with poorer health outcomes; this burden is especially acute for individuals with opioid use disorder (OUD), a chronic disease often associated with low socioeconomic status. Conventional travel time analyses may not fully account for experiential components of travel, thereby understating the true travel burden and overstating treatment accessibility to opioid treatment programs (OTPs).

**Objective:**

To develop a metric of feels-like accessibility for those using public transit to access OTPs that accounts for the realistic travel burden on individuals with OUD.

**Design, Setting, and Participants:**

This cross-sectional study integrated high-resolution transit schedules and operating hours of OTPs to measure feels-like accessibility. Feels-like accessibility considers the differential outcomes of out-of-vehicle travel components and more realistically reflects individuals’ transportation burden than conventional accessibility measures. Gini indices and spatial regression models were used to investigate inequities in accessibility. Geocoded data for residential addresses of 1018 overdose fatalities in Connecticut in 2019 were used as a proxy for the treatment needs of individuals with OUD. Data were analyzed between May and August 2023.

**Main Outcomes and Measures:**

Conventional and feels-like accessibility scores.

**Exposures:**

Fluctuations in public transit frequencies over the course of the day and the limited operating hours of the OTPs.

**Results:**

Of the 1018 individuals in the study, the mean (SD) age at death was 43.7 (12.6) years, 784 individuals (77%) were men, 111 (11%) were African American, and 889 (87%) were White, with other racial and ethnic categories including 18 individuals (2%). A total of 264 individuals in the sample (26%) could not access an OTP within 180 minutes. For those who could access these facilities, the average 1-way travel time was 45.6 minutes, with individuals spending approximately 70% of their trip duration on out-of-vehicle travel components. The conventional accessibility metric underestimates individuals’ travel burden to OTPs as well as the inequity in accessibility compared with the feels-like accessibility metric. For example, the median (range) conventional accessibility score, defined as the number of OTPs within 120 minutes of transit travel time, was 5.0 (0.0-17.0); the median (range) feels-like accessibility score, defined as the number of OTPs within 120 minutes of transit travel time weighted to account for in- and out-of-vehicle segments, was 1.0 (0.0-10.0). There is a considerable temporal variation in travel time and accessibility depending on the departure times.

**Conclusions and Relevance:**

In this cross-sectional study of travel burdens, the calculated feels-like accessibility scores, which consider the differential outcomes of out-of-vehicle travel components (eg, walking and waiting), could better and more realistically reflect passengers’ transportation burden. Policy recommendations derived from the conventional accessibility metric could be misleading, and decision-makers should use feels-like accessibility metrics that adequately capture individuals’ travel burdens. In the context of access to OTPs, the findings from this study suggest that opening new OTP sites to address gaps in access due to distance to services or extending hours of operation at existing sites may ameliorate the travel burden for individuals.

## Key Points

**Question:**

What are the travel burdens for individuals with opioid use disorder to access opioid treatment programs (OTP)?

**Findings:**

In this cross-sectional analysis of 1018 individuals, 26% of the sample could not access an OTP within 180 minutes. For those who could access these facilities, the average 1-way travel time was 45.6 minutes, with individuals spending approximately 70% of their trip duration on out-of-vehicle travel components.

**Meaning:**

These findings suggest that the conventional accessibility metric, which does not integrate out-of-vehicle (eg, walking or waiting) times, underestimates individuals’ travel burden to OTPs; public health policymakers should use feels-like accessibility metrics that adequately capture individuals’ true travel burdens.

## Teaser

This cross-sectional study evaluates accessibility for those using public transit to access opioid treatment programs in Connecticute while accounting for the realistic travel burden on individuals with opioid use disorder.

## Introduction

Transportation is a necessary step for accessing health care, particularly for people managing chronic diseases with their health care practitioners. Consequently, transportation barriers have long been associated with poorer health outcomes.^1^ This burden is especially acute for individuals with opioid use disorder (OUD), a chronic disease often associated with low socioeconomic status.^2,3,4^

This study examines the transportation obstacles in access to outpatient opioid treatment programs (OTPs) in Connecticut. We focus on OTPs as they are the exclusive source for methadone under current regulations. Prior studies examining transportation barriers for OUD have investigated differences in travel time by car or public transit between proxies for individual treatment need and treatment services.^5,6,7,8,9,10^ A few studies have demonstrated the impact of distance or travel time by car to OUD treatment and adverse outcomes, with 1 of these studies assessing the impact by individual demographics.^11,12,13^ However, these studies have not integrated the following 2 key features of travel burdens. First, the hours of operation in OTPs introduce constraints to accessing services and require awareness of the variation in transit schedules. Second, conventional travel time analyses may not fully account for experiential (feels-like) components of travel, thereby understating true travel burden. For example, getting to transit points from one’s residence and from a stop to the clinic can often entail lengthy walks. Waits at bus stops and train stations can add significant time and delay. In fact, those using public transportation perceive wait times to be between 1.5 to 4 times longer than they actually are.^14^

We sought to develop a more realistic understanding of transportation barriers for people with OUD, specifically for those traveling by public transit to OTPs, which often require daily presentation, especially during the early phases of methadone treatment. We focus on the use of public transportation since many individuals with OUD either do not own an automobile or may not have a valid driver’s license.^15^ Thus, automobile drive time alone cannot holistically capture travel burden for this population, and greater attention needs to be paid to public transportation.

In this study, we developed a novel metric of feels-like accessibility for those using public transit to access OTPs to account for the perceived travel burden on those seeking services. Specifically, we integrated transit schedules by bus and train into the analysis along with hours of operations of OTPs, which provides a way of understanding true accessibility to these services. We compared this feels-like accessibility with more conventional measures that simply considered total transit travel time. Next, we applied an equity lens to the feels-like accessibility to offer a better sense of the differential outcomes of travel burden by race and ethnicity, gender, age, and other covariates. Finally, we examined differences in treatment accessibility by time of departure due to variation in transit schedules and OTP hours of operation.

In legal settlements from the cases against opioid manufacturers, Connecticut will receive an estimated $600 million over 18 years for abatement efforts.^16^ One use for opioid settlement funds is expanding access to treatment for people with OUD. We hope this study will be able to inform where new services—in this case, OTPs—are needed in the state and how extended hours of operation and transportation access could improve access to medications for opioid use disorder. Since many states will receive these funds, we hope this research could be replicated in other settings to determine how to best improve access to methadone and combat the ongoing opioid epidemic.

## Methods

This research project has been reviewed by the Connecticut Office of the Chief Medical Examiner (OCME) and deemed not human participants research by the Yale University Human Investigations Committee, and as such informed consent was not required. While this is a descriptive modeling study and not a conventional cross-sectional analysis (ie, with outcomes and exposures), we have used the Strengthening the Reporting of Observational Studies in Epidemiology (STROBE) reporting guidelines for cross-sectional studies to guide the preparation of this manuscript.^17^

We used multiple sources of data to operationalize our algorithm, including information on people who need OUD treatment, treatment locations, and public transportation routes. For a deeper understanding of our data and methods, consult eAppendix 1 in Supplement 1.

Treatment need refers to those who have capacity to benefit from treatment.^18^ As a proxy for treatment need (ie, methadone), we used the 2019 data extracted from Connecticut’s OCME on accidental and undetermined overdose deaths that involved an opioid. We determined residential addresses in Connecticut for 1018 (89.4%) of the 1139 decedents. We excluded from our analysis any decedents that lacked identifiable Connecticut residential addresses in the OCME database (121 individuals [10.6%]). Specifically, we excluded decedents that either (1) had residential addresses outside of Connecticut (51 individuals [5.5%]), (2) had unmatchable residential addresses in Connecticut (7 individuals [0.6%]), or (3) had no residential address listed (63 individuals [5.5%]), of whom 41 (3.6%) were noted as homeless or transient, 4 (0.4%) as recently incarcerated, and 18 (1.6%) lacked any additional residence information. We chose those who died from opioid overdose as proxy participants for this analysis because easier access to methadone could have potentially decreased their risk for overdose (among other benefits).^19^ Data on decedents included their residential address (which we geocoded), age, sex, race and ethnicity as reported in the OCME data, population density, and neighborhood vulnerability levels (eAppendix 1 in Supplement 1).^20,21^ The race and ethnicity variable was included to examine the difference in travel time and accessibility scores among individuals’ race and ethnicity groups. Treatment locations consist of the 29 outpatient OTPs currently listed in the Methadone Treatment Programs database managed by the Connecticut State Department of Mental Health and Addiction Services.^22^

Using static General Transit Feed Specification datasets, we built a high-resolution, schedule-aware transit network for Connecticut.^23,24,25,26^ We computed an origin-destination transit travel time matrix. Using decedents’ residential locations as the starting point and each treatment facility as the destination, we calculated transit-based travel times for every origin-destination pair.^27^ We calculated individuals’ conventional transit-based accessibility scores to the OTPs. The conventional accessibility score is derived by counting the number of OTPs reachable within 120 minutes of transit travel time.

While useful, this conventional accessibility metric does not incorporate the discomfort experienced during in-vehicle vs out-of-vehicle (ie, walking or waiting) times, which potentially distort the reality individuals perceive depending on the distribution of their time spent in-vehicle vs out-of-vehicle. For example, for a transit trip of the same physical duration (say 60 minutes), person A who spends 60 minutes walking and waiting might perceive their journey as much longer than person B who spends 60 minutes within a moving vehicle. To address this limitation, we propose and use a feels-like accessibility measure. This measure provides a more realistic, user-centric portrayal of the actual transportation burden. Unlike the conventional accessibility score, the feels-like accessibility metric applies varying weights across in- and out-of-vehicle trip segments. Specifically, we assigned a weight factor of 2.0 to the out-of-vehicle component and 1.0 to the in-vehicle component based on findings from travel behavior studies.^28,29,30^ To investigate the inequality of accessibility, we computed the Gini indices. Because of variations in transit schedules and hours of operations of different OTPs across Connecticut, we explored the temporal variations of travel times using the feels-like accessibility. The analysis was repeated 6 times with different departure times. Lastly, we examined the extent to which the temporal variability of accessibility scores would be affected if there were no constraints on the operating hours of OTPs when computing accessibility.

### Statistical Analysis

Paired sample *t* tests were used to examine the differences in average conventional accessibility and feels-like accessibility scores. Spatial error models were also estimated to examine the associations between individuals’ accessibility scores and socioeconomic characteristics. A more detailed description of the model specification is provided in eAppendix 1 in Supplement 1. A 2-sided *P* value less than .05 is considered to be statistically significant. Data were analyzed between May and August 2023. Statistical analyses were performed using R version 4.3.0 (R Project for Statistical Computing).

## Results

### Travel Time to the Nearest OTP and Accessibility Scores

**Figure 1.**
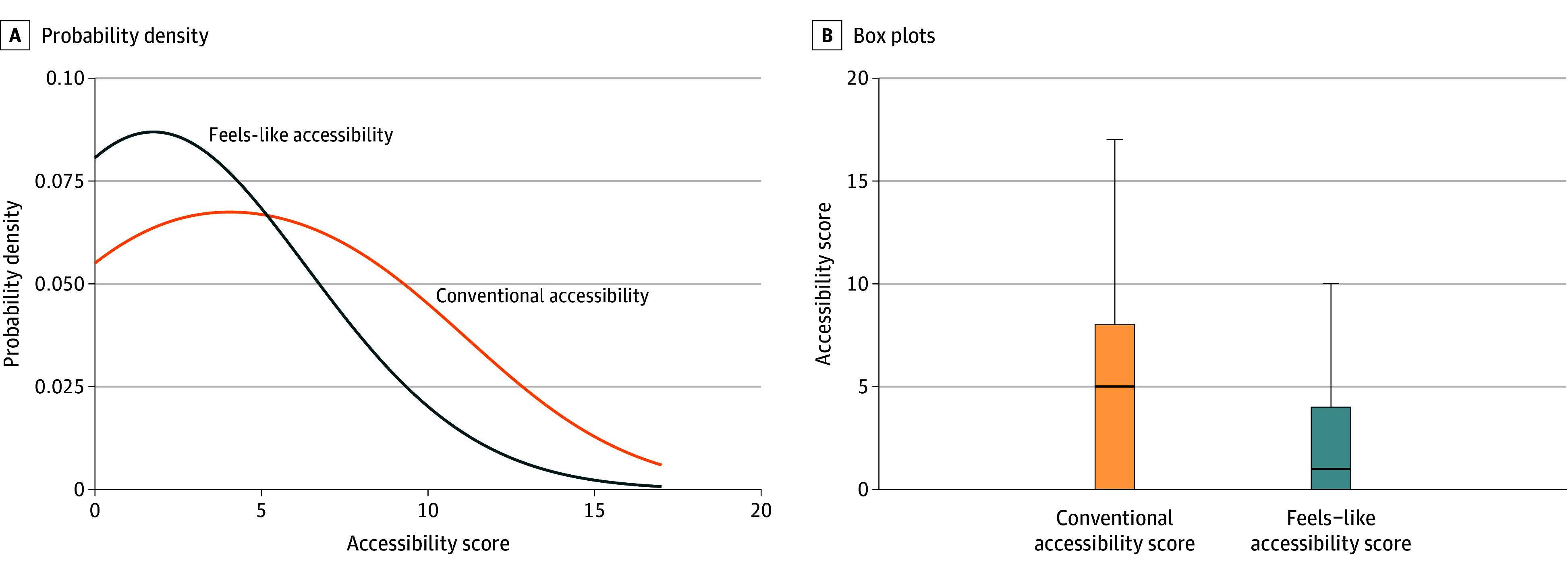
Results of Conventional and Feels-Like Accessibility Scores With 8 am Departure A, Probability density functions and B, box plots of transit-based conventional (orange) and feels-like accessibility scores (blue). The center lines inside the boxes indicate medians. The bottom and top borders of the boxes indicate the IQR. The whiskers denote 1.5 times the IQR.

Of the 1018 individuals in the study, the mean (SD) age at death was 43.7 (12.6) years, 784 individuals (77%) were men, 111 (11%) were African American, and 889 (87%) were White, with other racial and ethnic categories including 18 individuals (2%). Table 1 presents individuals’ transit travel time to the nearest OTP, focusing on 8 am as the departure time. Notably, approximately 26% of the 1018 individuals in the sample could not reach any facility via transit within 180 minutes. Among the remaining 74% who could access the facilities, the average 1-way transit travel time was 45.6 minutes. The breakdown of this statistic reveals that individuals spend an average of 16.7 minutes on access and egress walking, 13.6 minutes waiting, and 0.5 minutes walking between transfer points. This means that individuals spend approximately 70% of their trip duration on out-of-vehicle travel components. The average feels-like accessibility score is less than half of the average conventional accessibility score (Figure 1). The median (range) conventional accessibility score, defined as the number of OTPs within 120 minutes of transit travel time, was 5.0 (0.0-17.0); the median (range) feels-like accessibility score, defined as the number of OTPs within 120 minutes of transit travel time weighted to account for in- and out-of-vehicle segments, was 1.0 (0.0-10.0). The paired sample *t* test results show a significant difference between the 2 accessibility scores (*t*_1654.8_ = 17.128; *P* < .001).

**Table 1.** Public Transit-Based Travel Time and Conventional and Feels-Like Accessibility Scores^a^

Transit-based travel times and accessibility scores	Minutes	
	Median (range)	Mean (SD)
Total travel time	41.6 (0.9-160.3)	45.6 (25.3)
Access or egress walking time^b^	14.9 (0.8-54.4)	16.7 (10.6)
Waiting time	9.0 (0.0-109.9)	13.6 (14.8)
Transfer walking time	0.0 (0.0-16.1)	0.5 (1.5)
Conventional accessibility score^c^	5.0 (0.0-17.0)	4.5 (3.9)
Feels-like accessibility score^d^	1.0 (0.0-10.0)	2.1 (2.3)

^a^ Descriptive statistics of individuals’ transit-based travel time (1-way) to the closest opioid treatment program (OTP) and accessibility scores, assuming an 8 am departure, for individuals with less than 180 minutes travel time to the nearest OTP. The 2 formulations of the accessibility scores for these individuals are calculated from the different categories of travel time.

^b^ Access walking time indicates walking time from the origin (ie, home) to the closest transit station, and egress walking time indicates walking time from the final transit station to the destination (ie, OTP).

^c^ A conventional accessibility score is derived by counting the number of OTPs reachable within 120 minutes of transit travel time.

^d^ A feels-like accessibility metric applies varying weights across in- and out-of-vehicle trip segments.

### Inequality and Inequity in Accessibility Scores

The Gini indices for the conventional and the feels-like accessibility scores are 0.480 and 0.584, respectively, suggesting a higher degree of inequality in the feels-like accessibility scores compared with conventional accessibility scores (eAppendix 2 in Supplement 1). Table 2 presents the results of the spatial regression model. Age was significantly and positively associated with conventional accessibility scores but not with feels-like accessibility scores. Both population density and neighborhood vulnerability were also significantly and positively associated with both types of accessibility scores. However, the magnitude of both coefficients is smaller in model 2 than in model 1. Other variables did not exhibit significant associations in either model. These results suggest that increased population density and social vulnerability are associated with improved feels-like accessibility to OTPs, which is consistent with an increased concentration of transit stops in urban locales.

**Table 2.** Results of the Spatial Regression Models on the Associations Between Accessibility Scores and Sociodemographic Characteristics of Individuals^a^

Sociodemographic characteristic	Model 1: conventional accessibility score (SE)^b^	Model 2: feels-like accessibility score (SE)^c^
Age, y	0.009 (0.004)^d^	−0.001 (0.002)
Male (binary)	0.066 (0.125)	0.045 (0.066)
Other race and ethnicity (binary)^e^	−0.013 (0.174)	0.046 (0.091)
Ln Population density, person/m^2^^f^	0.547 (0.078)^g^	0.187 (0.042)^g^
Vulnerability level^h^	1.894 (0.383)^g^	1.043 (0.206)^g^
Constant	6.436 (0.758)^g^	2.575 (0.422)^g^
Observations, No.	1011	1011
Log likelihood	−2151.002	−1544.164
AIC	4318.003	3104.329

Abbreviation: AIC, Akaike information criterion.

^a^ Seven individuals (of 1018) who did not report the race variable were excluded from the models.

^b^ Conventional accessibility scores (model 1) represent the number of opioid treatment facilities accessible within 120 minutes of transit travel time.

^c^ Feels-like accessibility scores (model 2) represent the number of opioid treatment facilities accessible within 120 minutes of feels-like transit travel time.

^d^ *P* < .05.

^e^ Non-White group includes Asian, Black or African American, and other race. We used the binary group between White and non-White to pursue simplicity in interpreting model results.

^f^ Population density is measured at the census block group level.

^g^ *P* < .001.

^h^ Vulnerability level is measured at the census tract level. Vulnerability level ranges between 0 and 1.

### Temporal Variations in Travel Times and Accessibility Scores

**Figure 2.**
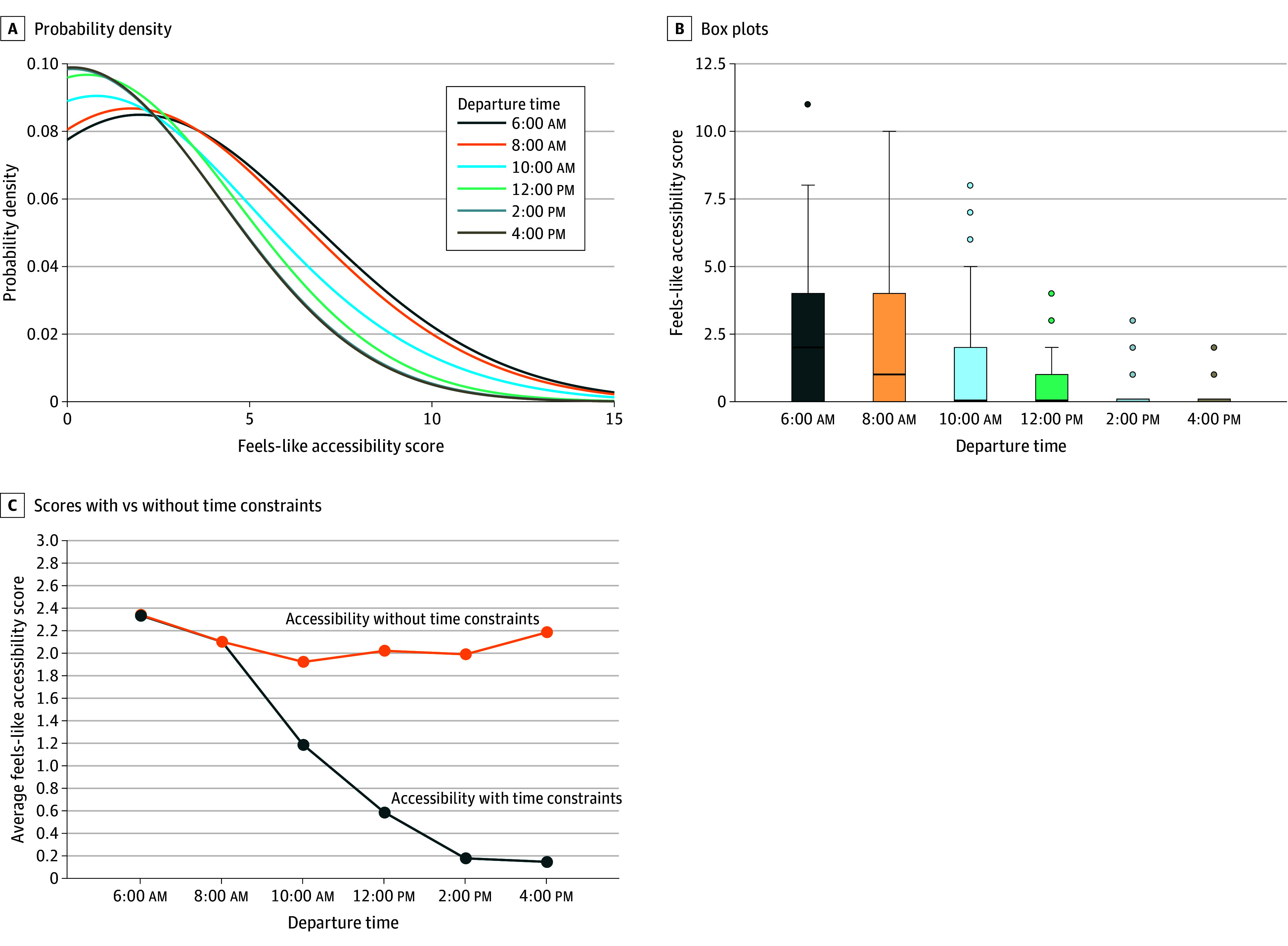
Feels-Like Accessibility Scores in Terms of Different Departure Times A, Probability density functions and B, box plots of individuals’ feels-like accessibility scores in terms of the different departure times. C, Comparison between feels-like accessibility scores with the consideration of operating hours (blue) and without the consideration of operating hours of opioid treatment programs (orange). The center lines inside the boxes indicate medians. The bottom and top borders of the boxes indicate the IQR. The whiskers denote 1.5 times the IQR. Dots indicate outliers.

In this section, we report the results on individuals’ travel time to the nearest OTP for different departure times (eAppendix 3 in Supplement 1). Overall, the average travel times for the afternoon trips were longer than those in the morning. For instance, the average travel time of the 4 pm trip was 71.6 minutes, which is about 1.4 times longer than the average travel time of the 6 am trip. There was a substantial temporal variation in feels-like accessibility scores. For instance, the average feels-like accessibility score of the 4 pm trip was 0.2, less than 10% of the average feels-like accessibility score of the 6 am trip (Figure 2A and 2B). We examined the extent to which the temporal variability of accessibility scores would be affected if there were no constraints on the operating hours of OTPs when computing accessibility (Figure 2C). The result shows that if there were no constraints on the operating hours of OTPs, accessibility scores do not substantially change in terms of the different departure times.

## Discussion

Our results revealed about 70% of transit trip time was spent on out-of-vehicle travel components. Given the negative outcomes of out-of-vehicle travel components on people’s disutility of transit trips, our findings are alarming because of the substantial travel burdens individuals face when attending OTPs.^28,29,30^ Travel burdens, especially early in treatment when daily visits to the OTP are required, are frequently cited as a reason for treatment drop-out.^8,11,31^ Of note, chronic pain is common in patients seeking and receiving methadone, ranging from 45% to 65%, as well as 40% in individuals with OUD who are not in any form of OUD treatment, which likely increases travel burden.^32,33,34^ The results also demonstrated that conventional accessibility metrics might underestimate the travel burden that individuals endure on their transit trips to treatment facilities as well as the inequality in transit-based accessibility to OTPs compared with the feels-like accessibility metric.

Our results underscored the considerable temporal variation in travel time and accessibility depending on the departure times. Accessibility is limited when individuals go in the afternoon rather than in the morning. While many OTPs open early in the morning to accommodate individuals before they set off to work, early closing times complicate treatment for individuals with nontraditional or irregular hours for work or other responsibilities (eg, childcare). Individuals with a history of OUD often find their best chances for employment are in such jobs.^35,36^ Considering the potentially limited time budget of socioeconomically disadvantaged people,^37,38^ our findings suggest that public health policies should aim to reduce this temporal variation. Two primary factors could contribute to this variation: fluctuations in public transit frequencies during the day and the limited operating hours of OTPs. Our analysis revealed that accessibility stayed at a similar level regardless of the different departure times. This suggests that constraints imposed by operating hours, rather than variations in the transit schedule, could be a primary factor in impeding access. Extending transit services can be more costly than extending the operating hours of OTPs. Therefore, our findings suggest that the temporal variation in accessibility can be effectively mitigated by extending the operating hours of treatment facilities.

### Limitations

Our study has several limitations that future studies can address. First, we chose the residential addresses of individuals who died from opioid overdose as a proxy for need of OTP services, which might not exactly reflect potential treatment need. Nevertheless, since it is difficult, if not impossible, to identify the underlying population with OUD that might benefit from OTP services, the data provided by the Connecticut OCME stands as the most reasonable proxy at our disposal. In addition, the decedents clearly represent a cohort that could have benefited from medications for opioid use disorder (MOUD). Second, by limiting our analysis to licensed OTPs, we are implicitly prioritizing methadone treatment over other medication treatments (eg, buprenorphine). Highlighting methadone access barriers and their role in the opioid overdose epidemic is in line with recommendations from national public health and policy experts and supplements other work focusing on MOUD accessibility.^11,39^ Future research on buprenorphine access might benefit from replicating the feels-like accessibility method used in the current study. Furthermore, there are policy measures that could ameliorate travel burdens in accessing methadone at OTPs by changing laws to allow primary care physicians and pharmacists to prescribe and dispense methadone.^40^ We have evaluated the provision of methadone by primary care physicians at Federally Qualified Health Centers (FHQCs) and dispensing at retail pharmacies (ie, at Walmart) in a previous article and new policy changes to make it possible for this to happen would likely reduce both conventional and feels-like accessibility for individuals.^5^ Third, we assumed that transit trips perfectly follow the schedule without any delays, which is unlikely in the real world where transit services are often delayed or disrupted. Future studies can consider collecting real-time transit operation data to examine accessibility based on the actual travel time that takes into account public transit unreliability.^41,42^ Fourth, our weight factor (2.0) is based on the literature on travel behavior. Future studies can calibrate the weight factor for feels-like accessibility to more realistically reflect the travel behavior of individuals with OUD living in Connecticut as well as other factors such as availability of real-time transit location information and seasonal weather variation (eg, waiting at bus stops on cold winter days vs warm spring ones).^43^ Fifth, in our mapping of treatment needs to Connecticut residential addresses, we have neglected to address access burdens among nonresidents, transient individuals, and the unhoused population, and without the location of these individuals it is impossible to calculate their travel times and burdens, limiting the generalizability of our results. Additionally, we did not perform a standard drive-time analysis by car in this study. Many drive time analyses have been conducted to evaluate access to MOUD, and we targeted transportation options that have been less explored in the literature but are more readily available to people with OUD. Public transit also represents a mode of transportation that could be seen as the floor for access—that is, the most time-consuming for individuals but most accessible and commonly used in many places in the US.

Our results imply that policy recommendations derived from the conventional accessibility metric can be misleading. The inequality in accessibility when evaluated using the conventional metric paints a rosier picture than the complicated reality of reliance on mass transit. The spatial regression models further highlighted the limitations of the conventional accessibility metric when formulating public health policies for promoting social equity. For instance, age had a significantly positive association with the conventional accessibility score but not with the feels-like accessibility score. In other words, younger age is significantly associated with lower conventional accessibility scores but not with the feels-like accessibility score, which more accurately represents the true travel burden. This suggests that if policymakers rely on the conventional accessibility metric, their recommended policies would favor younger people. However, this could be erroneous because age was not associated with the feels-like accessibility score. These findings underscore that public health policies should use transit-based accessibility metrics that adequately capture individuals’ travel burdens.

## Conclusion

In this cross-sectional study of travel burdens associated with access to OTPs, we have shown that a feels-like accessibility metric could more adequate address the true burdens facing individuals than conventional measures of transportation barriers to care. Our study has broad policy implications for improving access to methadone treatment for OUD and related services. Our analysis can be applied to states, counties, or other geographic locations to identify areas where treatment facilities are unavailable or difficult to access via public transit. This analysis can also be applied to improve access to other services related to OUD, such as buprenorphine prescribers, pharmacies, social service agencies, and harm reduction programs that are concentrated in urban centers. The analysis can guide decisions about locating mobile services or building new brick-and-mortar facilities for enhanced accessibility. Addressing limited and unequal access to treatment services is an important use of increased federal expenditures on harm reduction and OUD treatment and state allocation of opioid settlement funds. Determining how to best improve access to methadone through enhanced public transit services, extended OTP operating hours, the construction of new facilities, or the use of mobile methadone dispensing may lead to improved treatment compliance and public health outcomes.

## Notes

Supplement 1. **eAppendix 1. **More Information on the Research Methods **eAppendix 2. **Inequality in Accessibility Scores **eAppendix 3. **Temporal Variations in Travel Times and Accessibility Scores
eReferences

Supplement 2.
Data Sharing Statement
