# Supplementary material for: Accessibility of Opioid Treatment Programs Based on Conventional vs Perceived Travel Time Measures
Source: JAMA Netw Open. 2024 Feb 20;7(2):e240209. doi: 10.1001/jamanetworkopen.2024.0209 (PMC10879949; doi:10.1001/jamanetworkopen.2024.0209)
Supplement: Supplement 1. — eAppendix 1. More Information on the Research Methods eAppendix 2. Inequality in Accessibility Scores eAppendix 3. Temporal Variations in Travel Times and Accessibility Scores eReferences [file jamanetwopen-e240209-s001.pdf]

Supplemental Online Content

Kim J, Lee J, Thornhill TA, et al. Accessibility of opioid treatment programs based on conventional vs perceived travel time measures. *JAMA Netw Open*. 2024;7(2):e240209. doi:10.1001/jamanetworkopen.2024.0209

eAppendix 1. More Information on the Research Methods
eAppendix 2. Inequality in Accessibility Scores
eAppendix 3. Temporal Variations in Travel Times and Accessibility Scores
eReferences

This supplemental material has been provided by the authors to give readers additional information about their work.

© 2024 Kim J et al. *JAMA Network Open.*

eAppendix 1. More Information on the Research Methods

Data. Since the sample data do not include their socioeconomic information (e.g., income), we used the census tract-level CDC/ATSDR Social Vulnerability index as a proxy to measure individuals' socioeconomic status[1]. Census block group-based population densities are calculated based on the American Community Survey (ACS) 2019 5-year estimates data. **EFigure 1** illustrates the vulnerability level in the study area. As expected, vulnerability levels in urban centers (e.g., Hartford, New Haven, New London, and Waterbury) are higher than in other regions. **EFigure 1** also illustrates the location of transit stations and opioid treatment programs (OTPs) in the study area. These OTPs are a subset of all federally licensed OTPs in Connecticut and do not include OTPs that are either exclusively focused on withdrawal management or are restricted to specific populations (e.g., Veterans or incarcerated people). We obtained geographic coordinates and operating hours for the 29 methadone-dispensing OTPs operating in Connecticut.

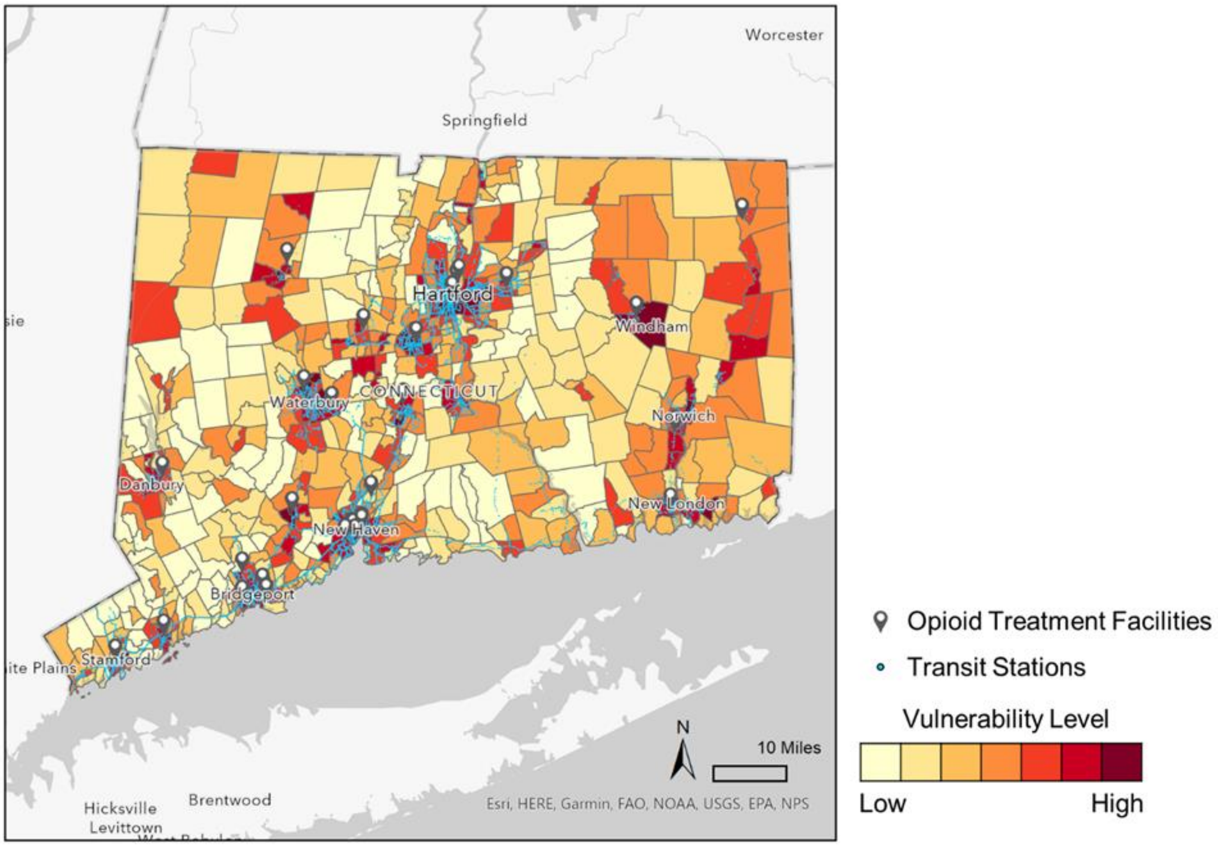

EFigure 1. A map showing the vulnerability level of census tracts, the location of transit stations, and the location of OTPs.

Computing and validating the transit travel time matrix. Four transit operators serving low-density regions did not provide GTFS data, so we manually created GTFS data for the latter four agencies based on their timetables and stop locations by using the National Rural Transit Assistance Program (RTAP) GTFS Builder as demonstrated in previous studies. We obtained

transit travel times (one-way) of all possible origin-destination pairs by using *r5r*, an open-source R-based transit analysis package. For more technical details of *r5r*, readers may refer to Pereira et al[2]. We set 30 minutes and 180 minutes for the maximum walking and total travel time, respectively. The departure date was set to be May 24, 2023 (Wednesday). To validate whether the *r5r* correctly computes transit travel times, we compared our travel times with those obtained from the Google Maps Distance Matrix API service. For validation purposes, we created 2000 by 2000 feet grid cells in Connecticut and used them for the departure locations. The destination was the Connecticut State Capitol, located in Hartford.

EFigure 2 compares the total travel times obtained from our *r5r* model with those obtained from Google Maps (2,799 origin-destination pairs). The figures show a strong consistency between the two travel time results. Moreover, the correlation coefficient was 0.765 (p<0.001), which confirms the validity of our travel time results. In what follows, **EFigures 3, 4**, and **5** illustrate access/egress walking time, waiting time, and the number of transfers of the trips, respectively.

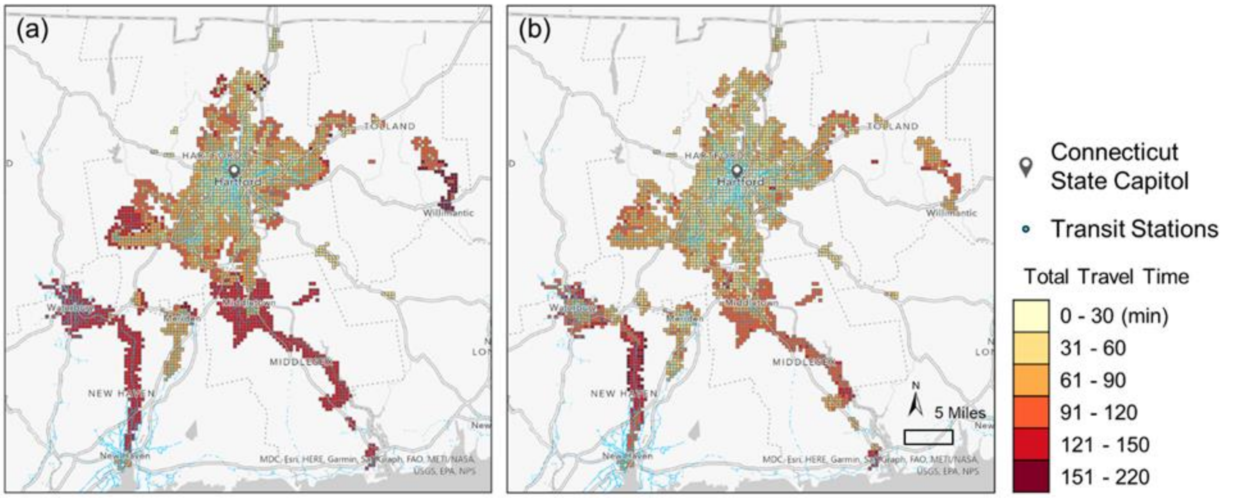

EFigure 2. A comparison between travel times obtained from (a) our *r5r* model and (b) Google Maps. Departure locations are grid cells (2000 by 2000 feet) in Connecticut, and the destination is the Connecticut State Capitol.

© 2024 Kim J et al. *JAMA Network Open.*

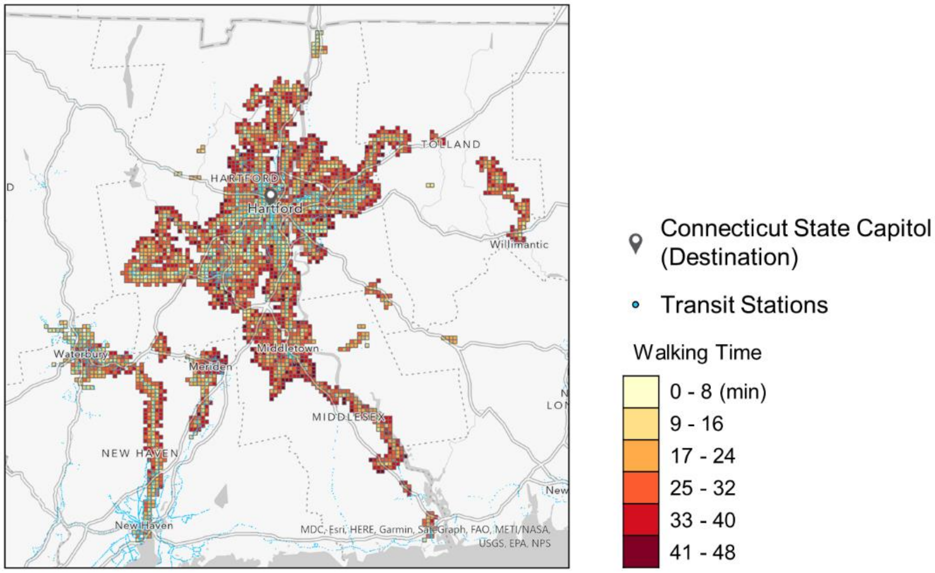

EFigure 3. Access/egress walking time of trips from the grid cells (2000 by 2000 feet) in Connecticut and to the Connecticut State Capitol.

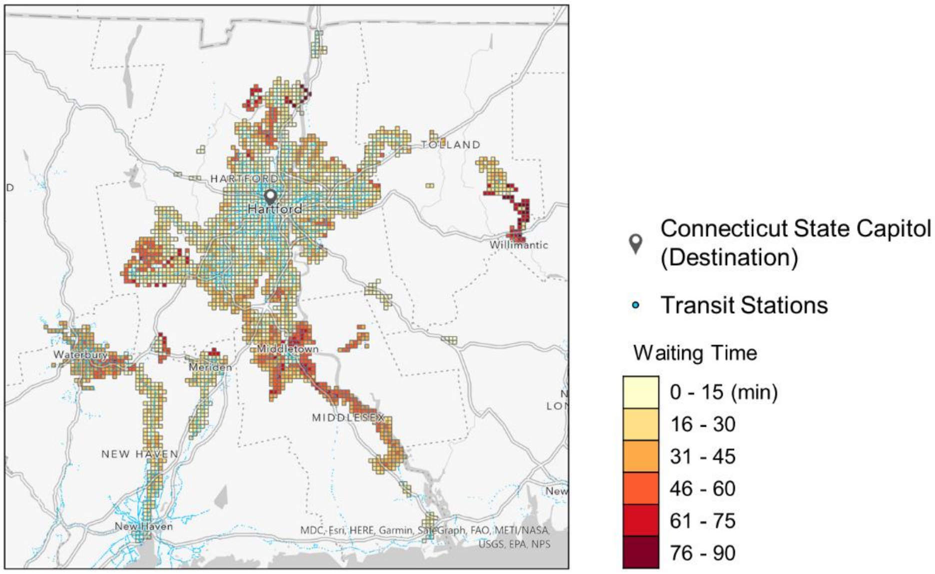

EFigure 4. Waiting time of trips from the grid cells (2000 by 2000 feet) in Connecticut and to the Connecticut State Capitol.

© 2024 Kim J et al. *JAMA Network Open.*

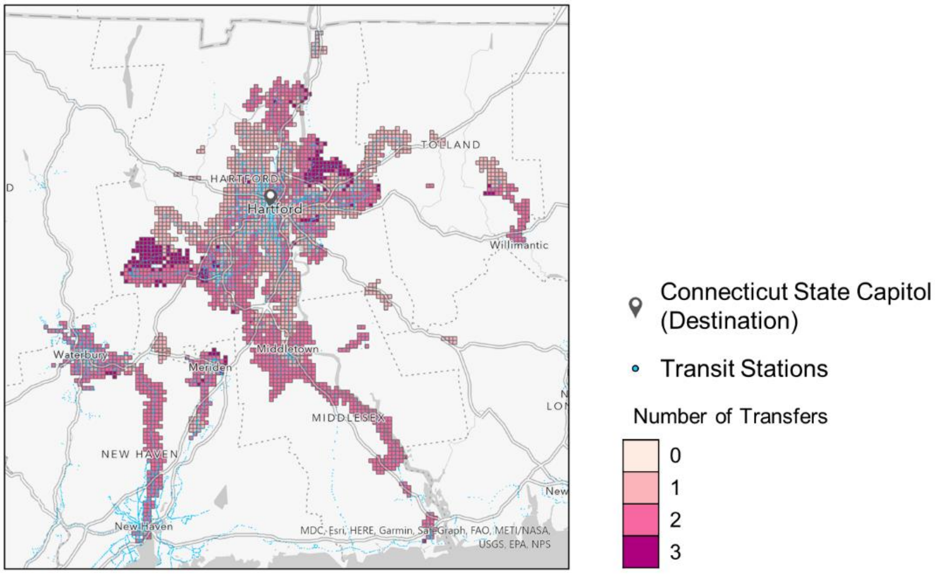

EFigure 5. The number of transfers of trips from the grid cells (2000 by 2000 feet) in Connecticut and to the Connecticut State Capitol.

Computing the accessibility scores. By using the computed transit travel time matrix, we computed individuals' transit-based accessibility to OTPs by using two metrics. Recall that the total transit travel time consists of access/egress walking, waiting, transfer walking, and riding time. The first metric is a conventional accessibility score, which is obtained by counting the number of OTPs that can be reached within 120 minutes of transit travel time (**Equation S1**).

$$CA_i = \sum_{j=1}^{N_{OTF}} C_j \ldots (S1)$$

, where CA_i is an individual i's ($i = 1,2,3, \ldots, 1{,}018$) conventional accessibility score, and N_{OTF} is the number of OTPs in Connecticut. C_j denotes 1 if OTP j is reached within 120 minutes transit travel time. Otherwise, C_j denotes 0.

The second metric is a feels-like accessibility score that better reflects the true travel burden individuals might experience compared to the conventional accessibility metric (**Equation S2**).

$$FA_i = \sum_{j=1}^{N_{OTF}} C_j \ldots (S2)$$

, where FA_i is an individual i's feels-like accessibility score. C_j denotes 1 if OTP is reached within 120 minutes feels-like transit travel time (**Equation S3**).

© 2024 Kim J et al. *JAMA Network Open.*

$$FTT_{i,j} = 2.0 \times (waiting\ time_{i,j} + access\ and\ egress\ walking\ time_{i,j} + transfer\ walking\ time_{i,j}) + 1.0 \times (riding\ time_{i,j}) \ldots (S3)$$

, where $FTT_{i,j}$ indicates feels-like transit travel time from individual i's home to facility j. Based on previous empirical travel behavior studies, we adopted 2.0 and 1.0 for the out-vehicle component and in-vehicle component weight factors, respectively [3–5].

Spatial regression model. Spatial regression models were estimated to examine the associations between individuals' accessibility scores and socioeconomic characteristics. We utilized the spatial regression model because of the presence of spatial autocorrelation in the residuals of the ordinary least squares (OLS) regression model. Specifically, the global Moran's I of the OLS residuals obtained through Monte-Carlo simulation with 999 permutations was 0.666 with a pseudo-p-value lower than 0.001. The spatial error model was selected based on the results of the Lagrange multiplier test[6]. **Equation S4** is the spatial error model that estimates the association between conventional accessibility score and sociodemographic variables.

$$CA_i = \beta_0 + \beta_1 Age_i + \beta_2 Male_i + \beta_3 Nonwhite_i + \beta_4 Populationdensity_i + \beta_5 Vulnerability_i + \lambda W u_i + \varepsilon_i \ldots (S4)$$

, where W is a spatial weight matrix and λ is the spatial lambda. u_i is a spatial component of the error term. ε_i is an independent identically distributed (IID) error term. The maximum likelihood (ML) estimation method is adopted by using the spdep r package (version 1.2.7)[7]. Similarly, **Equation S5** estimates the association between feels-like accessibility and sociodemographic variables.

$$FA_i = \beta_0 + \beta_1 Age_i + \beta_2 Male_i + \beta_3 Nonwhite_i + \beta_4 Populationdensity_i + \beta_5 Vulnerability_i + \lambda W u_i + \varepsilon_i \ldots (S5)$$

Regarding the spatial weight matrix, we used the K-nearest neighbor spatial weight matrices. We tested three different numbers of neighbors, including three, five, and seven. The core results (e.g., directions and significance levels of coefficients) remain consistent regardless of the selection of the spatial weight matrices (**ETables 1 and 2**). Thus, we have reported the spatial regression results based on the K-3 spatial weight matrix (**ETable 2**).

© 2024 Kim J et al. *JAMA Network Open.*

ETable 1. Results of the spatial error models on the associations between conventional accessibility scores and sociodemographic characteristics of individuals in terms of different spatial weight matrices.

Models	K-3 Spatial Weight Matrix			K-5 Spatial Weight Matrix			K-7 Spatial Weight Matrix		
Variables	Estimate	SE	p-value	Estimate	SE	p-value	Estimate	SE	p-value
Age	0.009	0.004	0.045	0.009	0.004	0.036	0.009	0.004	0.037
Male	0.066	0.125	0.598	0.023	0.129	0.856	-0.032	0.131	0.808
Non-white	-0.013	0.174	0.939	0.106	0.180	0.555	0.079	0.185	0.667
Pop. Density	0.547	0.078	<0.001	0.511	0.077	<0.001	0.492	0.076	<0.001
Vulnerability	1.894	0.383	<0.001	1.736	0.374	<0.001	1.622	0.360	<0.001
Constant	6.436	0.758	<0.001	6.312	0.781	<0.001	5.914	0.804	<0.001
Observations		1,011			1,011			1,011	
Log Likelihood		-2,151.002			-2,125.584			-2,120.626	
AIC		4,318.003			4,267.167			4,257.253	

ETable 2. Results of the spatial error models on the associations between feels-like accessibility scores and sociodemographic characteristics of individuals in terms of different spatial weight matrices.

Models	K-3 Spatial Weight Matrix			K-5 Spatial Weight Matrix			K-7 Spatial Weight Matrix		
Variables	Estimate	SE	p-value	Estimate	SE	p-value	Estimate	SE	p-value
Age	-0.001	0.002	0.641	-0.001	0.002	0.716	-0.001	0.002	0.762
Male	0.045	0.066	0.488	-0.002	0.069	0.972	-0.014	0.071	0.846
Non-white	0.046	0.091	0.616	0.150	0.096	0.119	0.146	0.100	0.145
Pop. Density	0.187	0.042	<0.001	0.172	0.041	<0.001	0.183	0.041	<0.001
Vulnerability	1.043	0.206	<0.001	1.050	0.202	<0.001	0.991	0.196	<0.001
Constant	2.575	0.422	<0.001	2.444	0.444	<0.001	2.305	0.460	<0.001
Observations		1,011			1,011			1,011	
Log Likelihood		-1,544.164			-1,512.981			-1,509.386	
AIC		3,104.329			3,041.961			3,034.771	

ETable 3. Descriptive statistics of the sample's demographic (n=1,018)

Demographics		Mean (SD) or Percentage
Age		43.694 (12.590)
Male		77.014 %
Race	African-American	10.904 %
	Asian	0.589 %
	Some Other Race	0.491 %
	White	87.328 %
	N/A	0.688 %

© 2024 Kim J et al. *JAMA Network Open.*

eAppendix 2. Inequality in Accessibility Scores

EFigure 6 illustrates Lorenz curves of conventional accessibility scores (red line) and feels-like accessibility scores (blue line). Recall that the Gini index obtained from the conventional accessibility scores (0.480) is lower than that obtained from the feels-like accessibility scores (0.584).

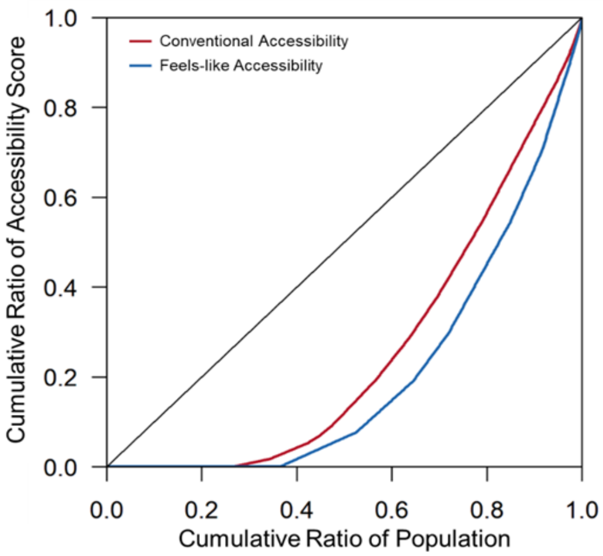

EFigure 6. Lorenz curves of individual scores of conventional (red) and feels-like (blue) transit-based accessibility to OTPs.

© 2024 Kim J et al. *JAMA Network Open.*

eAppendix 3. Temporal Variations in Travel Times and Accessibility Scores

ETable 4 illustrates the travel times and feels-like accessibility scores for different departure times. When individuals depart their trips at 6 AM, only 22% of them cannot make a transit-based trip to the closest treatment facility within a 180-minute travel time budget. This percentage becomes 85% for the 4 PM departure trips, which is approximately four times higher than the 6 AM departure time. In other words, more individuals cannot access OTPs when they make trips in the afternoon than in the morning.

ETable 4. Temporal variation of individuals' travel time to the closest OTP and feels-like accessibility scores in terms of the different departure times.

	Departure Time	Min (mins)	Median (mins)	Max (mins)	Mean (mins)	Standard Deviation
Travel Time	6 AM	0.9	41.8	171.3	49.9	30.9
	8 AM	0.9	41.6	160.3	45.6	25.3
	10 AM	0.9	60.3	174.8	73.9	43.4
	12 PM	0.9	47.6	154.2	60.6	37.7
	2 PM	6.3	97.2	175.5	97.6	46.7
	4 PM	9.3	81.8	144.2	71.6	34.6
	Departure Time	Min	Median	Max	Mean	Standard Deviation
Feels-like Accessibility	6 AM	0.0	2.0	11.0	2.3	2.5
	8 AM	0.0	1.0	10.0	2.1	2.3
	10 AM	0.0	0.0	8.0	1.2	2.0
	12 PM	0.0	0.0	4.0	0.6	1.0
	2 PM	0.0	0.0	3.0	0.2	0.7
	4 PM	0.0	0.0	2.0	0.2	0.5

© 2024 Kim J et al. *JAMA Network Open.*

eReferences

1. Centers for Disease Control and Prevention. CDC Social Vulnerability Index (SVI). Published September 4, 2020. Accessed September 16, 2020. https://www.atsdr.cdc.gov/placeandhealth/svi/index.html

2. Pereira RH, Saraiva M, Herszenhut D, Braga CKV, Conway MW. r5r: Rapid Realistic Routing on Multimodal Transport Networks with R 5 in R. *Findings*. Published online 2021.

3. Chapman B, Iseki H, Taylor BD, Miller M. The Effects of Out-of-Vehicle Time on Travel Behavior: Implications for Transit Transfers (Deliverable# 1). Published online 2006.

4. Guo Z, Wilson NH. Assessment of the transfer penalty for transit trips: Geographic information system-based disaggregate modeling approach. *Transportation Research Record*. 2004;(1872):10-18.

5. Kim J, Lee B. More than travel time: New accessibility index capturing the connectivity of transit services. *Journal of Transport Geography*. 2019;78:8-18.

6. Anselin, L. (1988). Lagrange multiplier test diagnostics for spatial dependence and spatial heterogeneity. *Geographical analysis*, 20(1), 1-17.

7. Roger Bivand (2022). “R Packages for Analyzing Spatial Data: A Comparative Case Study with Areal Data.” Geographical Analysis, 54(3), 488-518. doi:10.1111/gean.12319.

© 2024 Kim J et al. *JAMA Network Open.*
