# Supplementary material for: Accessibility of Opioid Treatment Programs Based on Conventional vs Perceived Travel Time Measures
Source: JAMA Netw Open. 2024 Feb 20;7(2):e240209. doi: 10.1001/jamanetworkopen.2024.0209 (PMC10879949; doi:10.1001/jamanetworkopen.2024.0209)
Supplement: Supplement 2. — Data Sharing Statement [file jamanetwopen-e240209-s002.pdf]

Data Sharing Statement

Kim. Accessibility of Opioid Treatment Programs Based on Conventional vs Perceived Travel Time Measures. *JAMA Netw Open*. Published February 22, 2024.
doi:10.1001/jamanetworkopen.2024.0209

Data
Data available: No

Additional Information
Explanation for why data not available: Data from the Connecticut's Office of the Chief Medical Examiner (OCME) on accidental and undetermined overdose deaths were obtained through a data use agreement, which precludes sharing this data publicly. However, it can be obtained by request to the Connecticut OCME.
